# Supplementary material for: Whole Genome Sequencing Applied in Familial Hamartomatous Polyposis Identifies Novel Structural Variations
Source: Genes (Basel). 2022 Aug 8;13(8):1408. doi: 10.3390/genes13081408 (PMC9407864; doi:10.3390/genes13081408)
Supplement: Supplementary file 1 [file genes-13-01408-s001.zip › Supplementary Table S1.pdf]

Supplementary Table S1: List of structural genetic alterations

Family	Main Genetic Alteration
A	INV BMPR1A Chr10:87,852,798-88,575,769 (723 Kb)
B	INV STK11 Chr19:1,206,071-1,274,737 (68 Kb)
C	BMPR1A del exons 3-13 (Chr10:88,611,032-88,964,753)
D	BMPR1A del exons 3-13 (Chr10:88,611,032-88,964,753)
E	BMPR1A c.1419delT, p.Val474Cysfs*24
